# Protocol for analyzing autophagy-related cellular phenotypes in autophagy-deficient human embryonic stem cell-derived lacrimal gland-like organoids

**DOI:** 10.1016/j.xpro.2026.104874

**Published:** 2026-10-01

**Authors:** Gamze Kocak, Sovan Sarkar

**Affiliations:** 1 Department of Cancer and Genomic Sciences, School of Medical Sciences, College of Medicine and Health, University of Birmingham, Birmingham B15 2TT, UK

**Keywords:** Cell Biology, Stem Cells, Organoids, Tissue Engineering

## Abstract

Autophagy is a vital catabolic process that maintains cellular homeostasis and survival and supports gland tissue development and function. Here, we present a protocol to characterize the cellular phenotypes of autophagy-deficient (*ATG5*^−/−^) lacrimal gland (LG)-like organoids derived from human embryonic stem cells. We describe steps for organoid cryosectioning, followed by the quantitative assessment of cell death and aggresomes using fluorescence-based TUNEL and ProteoStat staining, respectively, along with pharmacological rescue. This approach enables robust evaluation of autophagy-related cellular dysfunction in human organoid models.

For complete details on the use and execution of this protocol, please refer to Kocak et al.^1^

## Highlights

• Protocol for hESC-derived LG-like organoid culture and cryosectioning
• Analysis of cell death in hESC-derived LG-like organoids by TUNEL staining
• Analysis of aggresomes in hESC-derived LG-like organoids by ProteoStat staining
• Cellular phenotypes in *ATG5*^−/−^ LG-like organoids can be rescued by NMN treatment

## Abstract (editor highlights)

Publisher’s note: Undertaking any experimental protocol requires adherence to local institutional guidelines for laboratory safety and ethics.

## Graphical abstract

**Figure:**
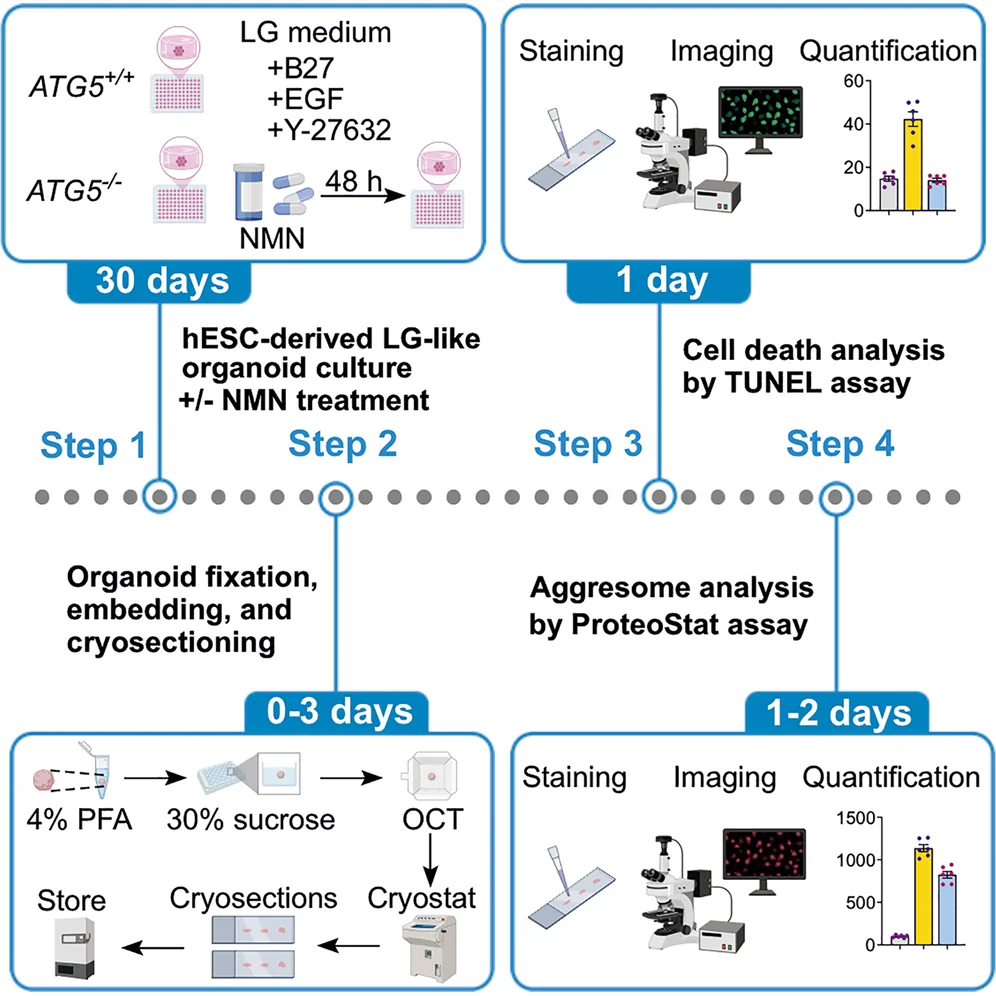

## Before you begin

In this protocol, we introduce a robust model to characterize key cellular phenotypes and evaluate pharmacological rescue using nicotinamide mononucleotide (NMN) in autophagy-deficient lacrimal gland (LG)-like organoids.

Wild-type (*ATG5*^+/+^) and autophagy-deficient (*ATG5*^−/−^)^2^ human embryonic stem cell (hESC)-derived LG-like organoids were generated by adapting previously established differentiation protocol.^1^^,^^3^ The methodology pertaining to the generation of LG-like organoids using multi-zonal ocular cell differentiation strategy, which is one of the strategies to mimic whole-eye development, is not included here.^1^^,^^3^^,^^4^^,^^5^ Instead, this protocol specifically details downstream phenotypic readouts and their pharmacological rescue in the autophagy-deficient LG-like organoid model to study the impact of autophagy deficiency on gland development.

Although optimized for LG-like organoids, this workflow can be readily adapted to other organoid systems to investigate autophagy-related cellular defects and evaluate pharmacological rescue strategies.

### Innovation

This protocol enables the modelling of autophagy deficiency in LG-like organoids and provides a robust framework for dissecting key cellular phenotypes, as well as evaluating pharmacological rescue strategies via NMN treatment. Compared with conventional two-dimensional (2D) culture systems, hESC-derived LG-like organoids provide a more physiologically relevant three-dimensional (3D) human model to investigate autophagy-related cellular dysfunction within a tissue-like context. The use of genetic hESC model of autophagy deficiency provides isogenic platform to study the consequences of autophagy deficiency in various differentiated cell-types and organoids, enabling quantitative assessment of phenotypic readouts pertaining to autophagy-related cellular dysfunction. For instance, the elevation of cell death and aggresomes observed in *ATG5*^−/−^ hESC-derived LG-like organoids were also found in our earlier study involving *ATG5*^−/−^ hESC-derived neurons.^2^ Our model enables mechanistic investigation of autophagy-related dysfunction and offers a platform for evaluating therapeutic interventions in a physiologically relevant *in vitro* organoid system. This approach can be adapted to other human pluripotent stem cell-derived organoid models for studying the homeostatic role and biomedical implications of autophagy.

### Institutional permissions

Users must obtain approval from their relevant institutional review boards or ethics committees prior to initiating experiments. All procedures should be conducted in accordance with applicable relevant institutional, national, and safety regulations. Researchers are responsible for ensuring that all experiments comply with approved protocols and relevant biosafety and regulatory standards.

### LG-like organoid maturation and culture

**Timing: 30 days**

The hESC lines used in this protocol include wild-type (*ATG5*^+/+^) cells and autophagy-deficient (*ATG5*^−/−^) cells.^2^ The autophagy-deficient hESC lines were previously generated from the parental WIBR3 hESC line^6^ through targeted deletion of exon 3 of an essential autophagy gene, *ATG5*, using transcription activator-like effector nucleases (TALENs).^2^ Loss of ATG5, which is required for autophagosome formation, prevents autophagy and thus its disruption is used to generate genetic knockout model of autophagy.^7^ Prior to differentiation, the hESC lines were maintained under feeder-free conditions on Geltrex basement membrane matrix. Methods for hESC characterization and generation of LG-like organoids are described in our original study.^1^ This method, adapted from a previous study,^3^ employs a multi-zonal ocular cell differentiation strategy followed by flow cytometric isolation of SSEA4/ITGB4 double-positive cells and subsequent embedding in Matrigel to generate LG-like organoids^1^ (Figure 1). In contrast, this protocol primarily focuses on the maturation of LG-like organoids, which takes 30 days, followed by downstream phenotypic characterization and pharmacological rescue approaches.

**Figure 1:**
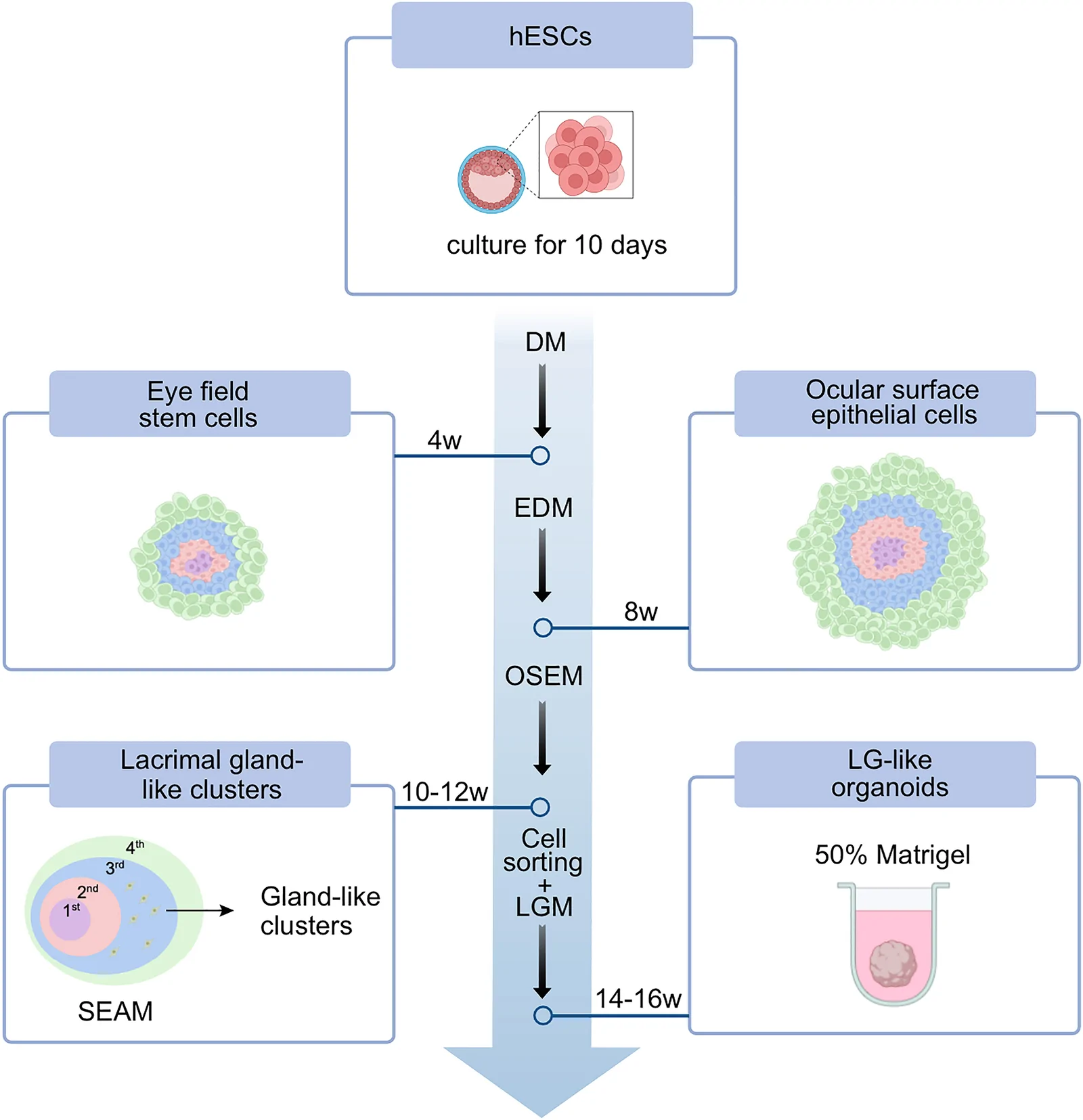
Schematic overview of the workflow for the generation and maintenance of human LG-like organoids hESCs were cultured for 10 days before being sequentially differentiated into SEAM through stage-specific medium changes, followed by isolation of LG progenitor marker-positive cells by cell sorting and generation of 3D LG-like organoids by embedding the sorted cells in 50% Matrigel. Representative morphology, culture media, and the timeline for each stage are illustrated. DM, differentiation medium; EDM, epithelial differentiation medium; OSEM, ocular surface epithelial differentiation medium; LGM, lacrimal gland culture medium.

In this protocol, LG-like organoids were generated from hESC-derived self-formed, ectodermal, autonomous, multi-zone (SEAM) cultures. Autophagy deficiency was confirmed by the absence of ATG5 (phagophore marker) and LC3-II (autophagosome marker) and the accumulation of p62 (autophagy substrate) in *ATG5*^−/−^ hESC-derived SEAM^1^ (Figure 2A). Mature LG-like organoids were further characterized by immunofluorescence staining for acinar/ductal (Claudin1, CK5, PANX1, and CK19) and myoepithelial (α-SMA) cell markers, confirming their LG lineage identity^1^ (Figure 2B).

1. Maintain LG-like organoid culture.
  a. The starting material is Matrigel-embedded immature three-dimensional LG-like organoids that are generated by the method described previously.^1^^,^^3^ These are cultured in ultra-low attachment, U-bottom, 96-well microplates where each well contains one organoid.
  b. Prepare LG culture medium as outlined in the materials and equipment section.
  c. Replace culture medium every 2–3 days for 30 days to generate mature LG-like organoids.

**Figure 2:**
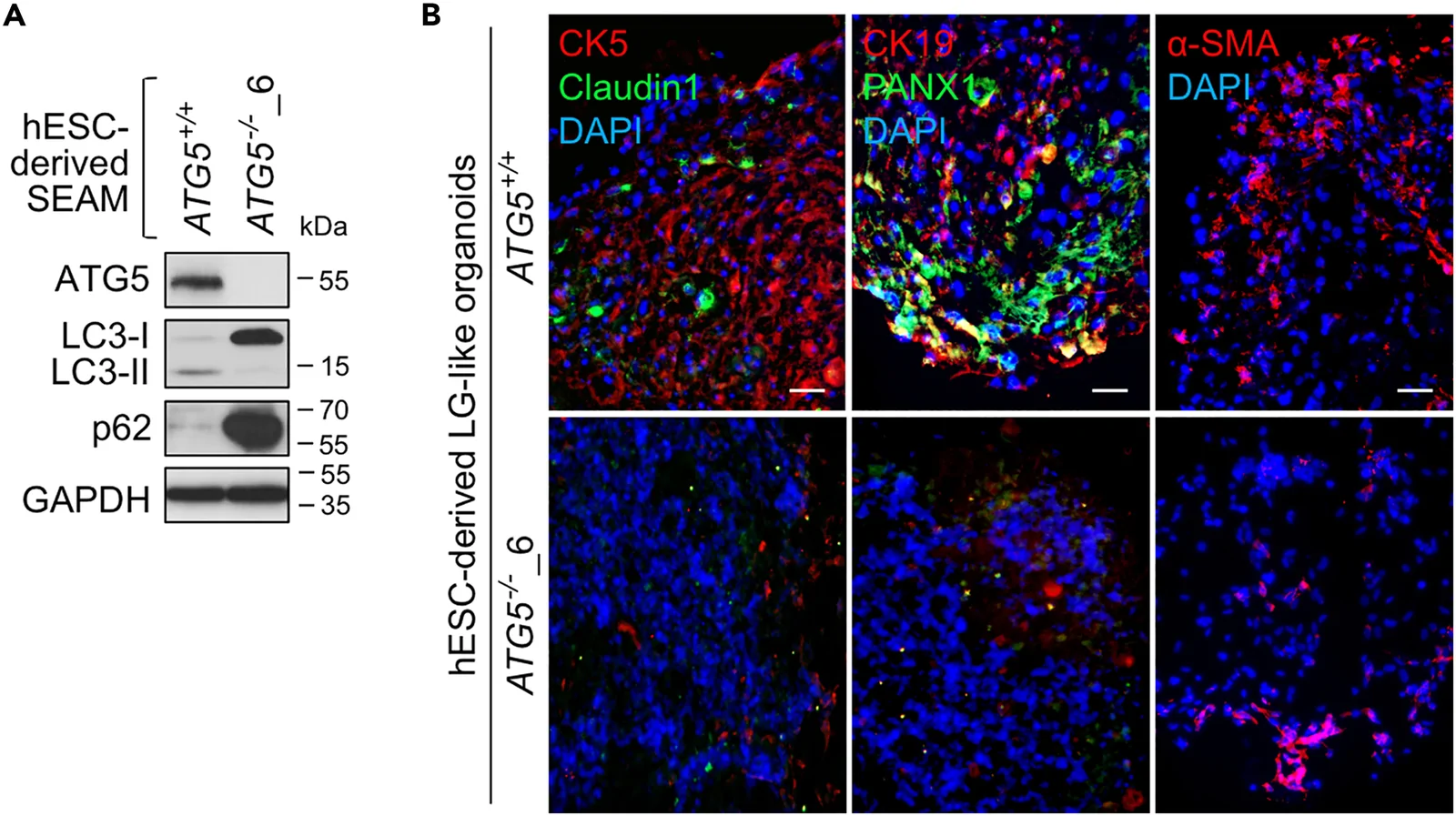
Characterization of autophagy and major cell-types in hESC-derived SEAM and LG-like organoids (A) Immunoblotting analyses of ATG5, p62, LC3 and GAPDH in *ATG5*^+/+^ and *ATG5*^−/−^_6 hESC-derived SEAM for characterization of loss of autophagy in autophagy-deficient SEAM. (B) Immunofluorescence images of CK5, Claudin1, CK19, PANX1 and α-SMA with DAPI nuclear staining in *ATG5*^+/+^ and *ATG5*^−/−^_6 hESC-derived LG-like organoids for characterization of major cell-types. Scale bar, 50 μm.

### NMN treatment

**Timing: 48 h**

We have recently demonstrated an evolutionarily conserved phenomenon that autophagy ensures cell survival by maintaining intracellular NAD pool (the oxidized NAD^+^ and reduced NADH forms), whereas loss of autophagy mediates cell death via NAD depletion. In autophagy-deficient cells or organisms, boosting NAD levels through supplementation of NAD^+^ precursors such as NMN improved cell viability and organismal survival.^2^^,^^8^ Therefore, NMN is used to rescue the cellular phenotypes in autophagy-deficient LG-like organoids.^1^

2. Prepare NMN solution.
  a. Prepare 100 mM NMN stock solution as outlined in the materials and equipment section.
3. Apply NMN to organoids.
  a. After 30 days of culture, remove the culture medium from the wells of 96-well microplates and replace it with fresh medium in both *ATG5*^+/+^ and *ATG5*^−/−^ LG-like organoids (for untreated conditions), or supplemented with 1 mM NMN in *ATG5*^−/−^ LG-like organoids.
  b. After 24 h, replace the medium with freshly prepared 1 mM NMN-supplemented medium in *ATG5*^−/−^ LG-like organoids and continue incubation for an additional 24 h. Also replenish the untreated conditions with fresh medium without NMN.

**CRITICAL:** NMN solutions should be prepared fresh immediately before use to ensure its stability and biological activity.

***Note:*** Treatment duration may require optimization depending on the organoid type, developmental stage, and specific experimental conditions.

### Organoid fixation, embedding, and cryosectioning

**Timing: 0–3 days**

4. Organoid fixation.
  a. Prepare fixation buffer (4% paraformaldehyde in 1× PBS) as outlined in the materials and equipment section.
  b. Using wide-bore pipette tips prepared by cutting standard tips with scissors, transfer each organoid from the wells of the 96-well microplate into separate labeled 1.5 mL Eppendorf tubes, ensuring one organoid per tube.
  c. Wash the organoids with ice-cold 1× PBS, keeping them on ice, until all residual Matrigel is completely removed.
  d. Fix organoids in 500 μL of fixation buffer per organoid for overnight at 4°C.

**CRITICAL:** Maintain precise fixation times. Inadequate or excessive fixation can destroy tissue morphology, leading to suboptimal staining and imaging outcomes.

***Note:*** Fixation parameters, including type of fixative, incubation time, and temperature, must be optimized for each organoid model.

5. Organoid embedding.
  a. Remove fixation buffer from the Eppendorf tubes and rinse organoids with 1× PBS three times for 5 min each.
  b. Using wide-bore pipette tips, transfer each organoid to an individual well of a 24-well plate containing 1–2 mL of 30% sucrose in 1× PBS per well (as outlined in the materials and equipment section) for overnight at 4°C until they sink.
    ***Alternatives:*** Organoids can be left in 30% sucrose at 4°C for up to 3 days. To prevent evaporation and/or contamination, tightly seal the Eppendorf tube with Parafilm.
  c. Fully remove the sucrose solution from the 24-well plate containing organoids.
  d. Transfer organoids to a cryomold containing OCT (Optimal Cutting Temperature) compound using wide-bore pipette tips (see key resources table).
    ***Alternatives:*** Cryomolds are commercially available; however, objects of an appropriate size can also be used as improvised molds depending on the organoid size; for example, the lid of an Eppendorf tube.
    **CRITICAL:** Make sure the organoids are fully immersed in OCT without air bubbles.
    ***Note:*** Prevent the formation of bubbles in the OCT, especially surrounding the organoid, as these can compromise sectioning quality.
  e. Immediately transfer the samples to −80°C freezer for rapid freezing until solid.
    **CRITICAL:** Be fast, as slow freezing promotes ice crystal formation.
    ***Alternatives***: Prepare dry ice cooling bath by filling a box with dry ice and placing a glass beaker on top of the dry ice.
  f. Store the organoid blocks at −80°C freezer.
    ***Note:*** Frozen organoids can be stored at −80°C for long-term preservation.
6. Organoid cryosectioning.
  a. Transport the OCT-embedded organoids on dry ice to a cryostat.
  b. Set up the cryostat as outlined in the materials and equipment section.
    **CRITICAL:** Organoids must remain completely frozen at all times. Do not allow the block to thaw or soften.
  c. Cut sections according to the desired thickness.
    **CRITICAL:** Be cautions during sectioning as cryostat blades are very sharp.
    ***Note:*** Use a brush to flatten the emerging section.
  d. Transfer the cryosections gently onto adhesive slides.
  e. Store sectioned organoids at −80°C freezer until staining.

## Key resources table

**Table:** 

REAGENT or RESOURCE	SOURCE	IDENTIFIER
**Antibodies**		

α-SMA monoclonal antibody (mouse) 1:200 dilution for immunofluorescence	Cell Signaling Technology	Cat # 48938S
ATG5 monoclonal antibody (mouse) 1:500 dilution for immunoblotting	Santa Cruz Biotechnology	Cat # sc-133158
CK5 monoclonal antibody (mouse) 1:200 dilution for immunofluorescence	Thermo Scientific	Cat # MA517057
CK19 monoclonal antibody (mouse) 1:200 dilution for immunofluorescence	Cell Signaling Technology	Cat # 4558S
Claudin1 monoclonal antibody (rabbit) 1:200 dilution for immunofluorescence	Cell Signaling Technology	Cat # 13995S
LC3B polyclonal antibody (rabbit) 1:2000 dilution for immunoblotting	Proteintech	Cat # 14600-1-AP
PANX1 monoclonal antibody (rabbit) 1:100 dilution for immunofluorescence	Sigma-Aldrich	Cat # HPA016930
p62 polyclonal antibody (mouse) 1:3000 dilution for immunoblotting	Proteintech	Cat # 66184-1-Ig
Goat anti-rabbit IgG, H&L chain specific peroxidase conjugate; 1:10000 dilution for immunoblotting	Dako	Cat # P0448
Rabbit anti-mouse IgG, H&L chain specific peroxidase conjugate; 1:10000 dilution for immunoblotting	Dako	Cat # P0161
Donkey anti-mouse IgG (H + L), Alexa Fluor 594; 1:1000 dilution for immunofluorescence	Thermo Scientific	Cat # A21203
Donkey anti-rabbit IgG (H + L), Alexa Fluor 488; 1:1000 dilution for immunofluorescence	Thermo Scientific	Cat # A21206

**Chemicals, peptides, and recombinant proteins**		

Animal-Free Recombinant Human EGF	PeproTech	Cat# AF-100–15
B27 plus supplement (50×)	Gibco	Cat# 12587010
Bovine Serum Albumin (BSA)	Sigma-Aldrich	Cat# A7030
DMEM/F12	Gibco	Cat# 11320033
Dulbecco’s Phosphate Buffered Saline (PBS)	Gibco	Cat#14190094
Nicotinamide mononucleotide (NMN)	NMN Bio	Cat# NMN01-16676
OCT compound	Epredia	Cat# 11912365
Paraformaldehyde Solution 16%	Thermo Scientific	Cat# 15670799
Penicillin-Streptomycin (5000 U/ml)	Gibco	Cat# 15070063
ProLong Gold Antifade Mountant with DAPI	Invitrogen	Cat# P36931
ProLong Gold Antifade Mountant	Invitrogen	Cat# P36930
Sucrose	Sigma-Aldrich	Cat# S9378
Triton X-100	Thermo Scientific	Cat# 9036–19–5
Y-27632 dihydrochloride	Tocris	Cat# 1254

**Critical commercial assays**		

Click-iT Plus TUNEL Assay Kit	Invitrogen	Cat# C10617
PROTEOSTAT Aggresome Detection Kit	Enzo	Cat# ENZ-51035-K100

**Experimental models: Cell lines**		

*ATG5*^+/+^ (WIBR3) hESCs	Lengner et al.^6^	N/A
*ATG5*^−/−^ (clone #6) hESCs	Sun et al.^2^	N/A
hESC-derived LG-like organoids	Kocak et al.^1^	N/A

**Software and algorithms**		

GraphPad Prism v9.5.0	GraphPad Software	https://www.graphpad.com/; RRID: SCR_002798
ImageJ v2.9.0	NIH	https://imagej.net/ij/index.html; RRID:SCR_003070

**Other**		

24-well plates	Corning	Cat# 10380932
96-well U-bottom ultra-low attachment microplates	Corning	Cat# 10023683
Cryostat	Bright	OTF5000
Disposable Microtome Blades	Feather S35	N/A
EVOS FL Fluorescence Microscope	ThermoFisher Scientific	N/A
Hydrophobic Barrier PAP Pen	Sigma-Aldrich	Cat# Z672548-1EA
Microtube (1.5 mL)	Eppendorf	Cat# 022364111
Superfrost Plus adhesion microscope slides	Epredia	Cat# 10149870

## Materials and equipment

***Note:*** Store at 4°C for up to 1 month.

**Table:** Lacrimal gland (LG)-like organoid culture medium

Reagent	Final concentration	Amount
DMEM/F12	N/A	24.220 mL
B27 plus supplement (50×)	2%	500 μL
Animal-Free Recombinant Human EGF (100 μg/mL)	20 ng/mL	5 μL
Penicillin-Streptomycin (5000 U/mL)	50 U/mL	250 μL
Y-27632 (10 mM)	10 μM	25 μL
**Total**	**N/A**	**25 mL**

**Table:** NMN stock solution

Reagent	Final concentration	Amount
NMN	100 mM	16.711 mg
dH_2_O	N/A	500 μL
**Total**	**N/A**	**500 μL**

***Note:*** Prepare fresh and filter with 0.22 μm sterile syringe filter.

**Table:** Fixative solution

Reagent	Final concentration	Amount
16% Paraformaldehyde	4%	10 mL
PBS	N/A	To 50 mL
**Total**	**N/A**	**50 mL**

***Note:*** Store in aliquots at −20°C for up to 6 months. Avoid repeated freeze-thaw cycles.

**CRITICAL:** Paraformaldehyde is hazardous. Handle only in a fume hood and dispose of it in accordance with institute regulations.

**Table:** 30% sucrose solution

Reagent	Final concentration	Amount
Sucrose	30%	30 g
PBS	N/A	To 100 mL
**Total**	**N/A**	**100 mL**

***Note:*** Store at 4°C for up to 2 weeks or −20°C for up to 6 months.

**Table:** Permeabilization solution

Reagent	Final concentration	Amount
Triton™X-100	0.25%–0.5%	25 μL–50 μL
PBS	N/A	To 10 mL
**Total**	**N/A**	**10 mL**

***Note:*** Store at 4°C for up to 2 weeks.

**Table:** 3% BSA solution

Reagent	Final concentration	Amount
Bovine serum albumin (BSA)	3%	0.3 g
PBS	N/A	To 10 mL
**Total**	**N/A**	**10 mL**

***Note:*** Prepare fresh.

**Cryosectioning settings:** Set the cryostat chamber temperature between −15°C and −20°C. In this protocol, sections are cut at a thickness of 8 μm.

***Note:*** Permeabilization times should be optimized according to the section thickness.

## Step-by-step method details

### Cell death analysis by TUNEL assay

**Timing: 1 day**

To evaluate the impact of autophagy deficiency on cell viability in LG-like organoids and to determine the cytoprotective effect of NMN treatment, cell death is analyzed in the organoid cryosections using the Click-iT Plus TUNEL Assay for In Situ Apoptosis Detection (Invitrogen) (see key resources table). This assay labels damaged DNA containing exposed 3′-OH ends, allowing detection of apoptotic nuclei by fluorescence microscopy.^9^^,^^10^ TUNEL^+^ apoptotic nuclei are quantified in cryosections of *ATG5*^+/+^ and *ATG5*^−/−^ hESC-derived LG-like organoids, including NMN-treated *ATG5*^−/−^ organoids^1^ (Figures 3A and 3B).

1. Prepare LG-like organoid cryosections for assay.
  a. Remove cryosection slides from −80°C storage and thaw at room temperature for 20 min.
  b. Draw a hydrophobic barrier by surround each cryosection using a PAP pen. Approximately 3–4 cryosections are there on each slide.
  c. Wash the slides by gently adding 20–30 μL of 1× PBS to each cryosection surrounded by hydrophobic barrier, three times for 5 min each.
2. Perform TUNEL assay using Click-iT Plus TUNEL Assay Kit, Alexa Flour 488 dye (Invitrogen).
  a. Prepare the working solutions using the kit components according to manufacturer’s instructions.
  b. Permeabilize sections with 20–30 μL of 0.25% permeabilization buffer per section and incubate 20 min at room temperature.
  c. Wash the slides by gently adding 20–30 μL of 1× PBS to each cryosection surrounded by hydrophobic barrier, three times for 5 min each.
  d. Add 20–30 μL of terminal deoxynucleotidyl transferase (TdT) Reaction Buffer per section and incubate for 10 min at 37°C in a humidified incubator.
  e. Prepare TdT reaction mixtures using the kit components according to manufacturer’s instructions.
  f. Remove TdT Reaction Buffer, add 20–30 μL TdT reaction mixture to each section and incubate for 60 min at 37°C in a humidified incubator.
  g. Remove TdT reaction mixture, wash the slide with 3% Bovine Serum Albumin (BSA) in 1× PBS for 5 min.
  h. Rinse the cryosection slides once with 1× PBS.
  i. Prepare Click-iT Plus TUNEL Buffer Additive using the kit components according to manufacturer’s instructions.
  j. Prepare Click-iT Plus TUNEL reaction cocktail using the kit components with Click-iT Plus TUNEL Buffer Additive according to manufacturer’s instructions.
  k. Immediately add 20–30 μL Click-iT Plus TUNEL reaction cocktail to each section and incubate for 30 min at 37°C in a humidified incubator, protect from light.
  l. Remove Click-iT Plus TUNEL reaction cocktail, then wash the slide with 3% Bovine BSA in 1× PBS for 5 min.
  m. Rinse the cryosection slides once with 1× PBS.
    ***Note:*** Adjust the volume of the solutions depending on the section size, protected from light.
    ***Note:*** Use humidified chamber during the staining procedures to avoid evaporation.
  n. Mount each slide with coverslip using ProLong Gold antifade reagent with DAPI and incubate at room temperature in the dark for 15–30 min.
  o. Seal coverslip onto glass slides using nail varnish, taking care not to move the coverslips on the surface of glass slides.
    ***Alternatives***: Sections can be blocked with 3% BSA in 1× PBS and subjected to immunostaining for additional markers if required.
    **CRITICAL:** Cryosections must not be allowed to dry during the staining procedures because this can significantly reduce signal intensity.
3. Analyze TUNEL^+^ apoptotic nuclei by fluorescence microscopy.
  a. Acquire fluorescence images using appropriate filter sets for Alexa Flour 488 picolyl azide dye (TUNEL) and DAPI.
  b. Assess the number of TUNEL^+^ apoptotic nuclei and analyze ∼400–500 cells per sample.
  c. Calculate the percentage of TUNEL^+^ apoptotic nuclei relative to the total number of cells analyzed for each sample.

**Figure 3:**
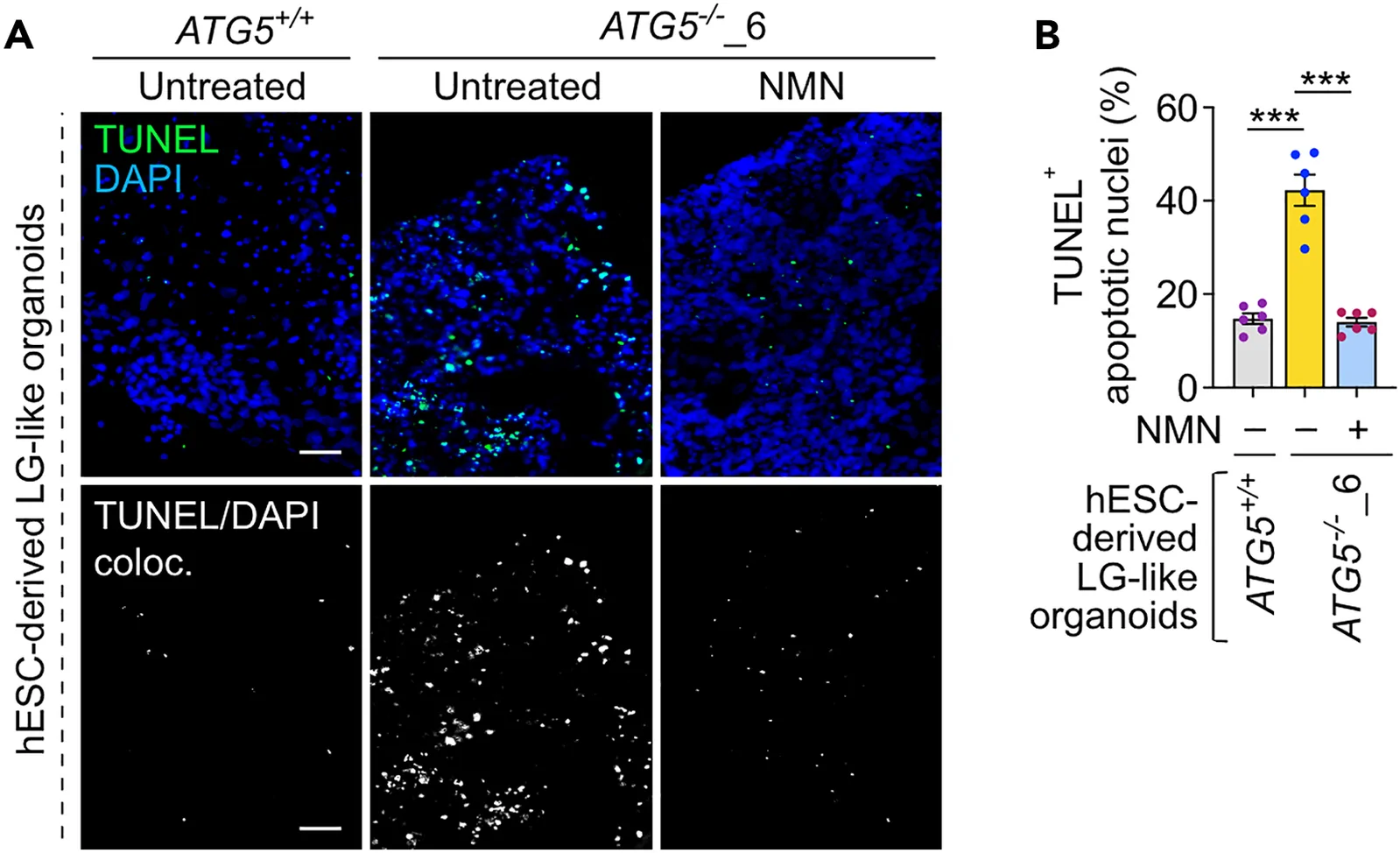
Elevated cell death in autophagy-deficient hESC-derived LG-like organoids is rescued by NMN treatment (A and B) Fluorescence images of TUNEL staining (A) and quantification of TUNEL^+^ apoptotic nuclei (B) in *ATG5*^+/+^ and *ATG5*^−/−^_6 hESC-derived LG-like organoids, where *ATG5*^−/−^ organoids were treated with or without 1 mM NMN for 48 h. Graphical data is represented as mean ± SEM of n = 6 experimental replicates from 3 independent experiments (B). *P* values are calculated by one-way ANOVA followed by multiple comparisons with a two-stage linear step-up procedure of Benjamini, Krieger and Yekutieli. ∗∗∗P<0.001. Scale bar, 50 μm (A).

### Aggresome accumulation analysis by ProteoStat assay

**Timing: 1–2 days**

To evaluate the effects of autophagy deficiency on proteostasis in LG-like organoids and to determine the potential cytoprotective effect of NMN treatment, aggresomes (intracellular inclusion bodies composed of aggregated, misfolded proteins) are examined in organoid cryosections using the PROTEOSTAT® Aggresome Detection kit (Enzo) (see key resources table). This assay utilizes a red-fluorescent molecular rotor dye that selectively binds to the cross-β sheet structures characteristic of protein aggregates, enabling visualization and quantitative analysis of aggresomes within cells.^11^^,^^12^ ProteoStat fluorescence intensity is quantified in cryosections of *ATG5*^+/+^ and *ATG5*^−/−^ hESC-derived LG-like organoids, including NMN-treated *ATG5*^−/−^ organoids^1^ (Figures 4A and 4B).

4. Perform ProteoStat assay using PROTEOSTAT® Aggresome Detection kit (Enzo).
  a. Cryosection slides are prepared and processed as described in step 1 of TUNEL assay protocol, followed by ProteoStat staining as described below.
  b. Permeabilize sections with 20–30 μL of 0.5% permeabilization buffer per section and incubate 1 h at room temperature.
  c. Wash the slides by gently adding 20–30 μL of 1× PBS to each cryosection surrounded by hydrophobic barrier, three times for 5 min each.
  d. Prepare 1× Assay Buffer according to manufacturer’s instruction.
  e. Prepare the Dual Detection Reagent with ProteoStat Aggresome Dye (1:2000 dilution) and Hoechst 33342 nuclear stain (1:1000 dilution) in 1 × Assay Buffer.
  f. Add 20–30 μL of the Dual Detection Reagent each section and incubate overnight at 37°C in humidified chamber protected from light.
  g. Remove the Dual Detection Reagent, wash the slide with 1× PBS three times for 5 min each as mentioned above.
    ***Note:*** Adjust staining volumes according to section size and protect from light throughout the whole procedure.
  h. Mount each slide with coverslip using ProLong Gold antifade reagent (without DAPI) and incubate at room temperature in the dark for 15–30 min.
  i. Seal coverslip onto glass slides using nail varnish, taking care not to move the coverslips on the surface of glass slides.
5. Analyze the stained cells by fluorescence microscopy.
  a. Acquire fluorescence images using appropriate filter sets for PROTEOSTAT® Aggresome dye (excitation/emission 500/600 nm, Texas Red channel) and Hoechst 33342 (DAPI channel).
  b. Assess the ProteoStat fluorescence intensity using Fiji/ImageJ software and analyze ∼400–500 cells per sample.
  c. Measure the integrated density and number of nuclei analyzed for each sample, and calculate fluorescence as integrated density per cell.
  d. Calculate the relative ProteoStat fluorescence intensity of all samples by normalizing the respective integrated density per cell values to the mean integrated density of the *ATG5*^+/+^ LG-like organoids (wild-type or control condition). The normalized data are presented as percentage relative to the control condition, which is set to 100%.

**Figure 4:**
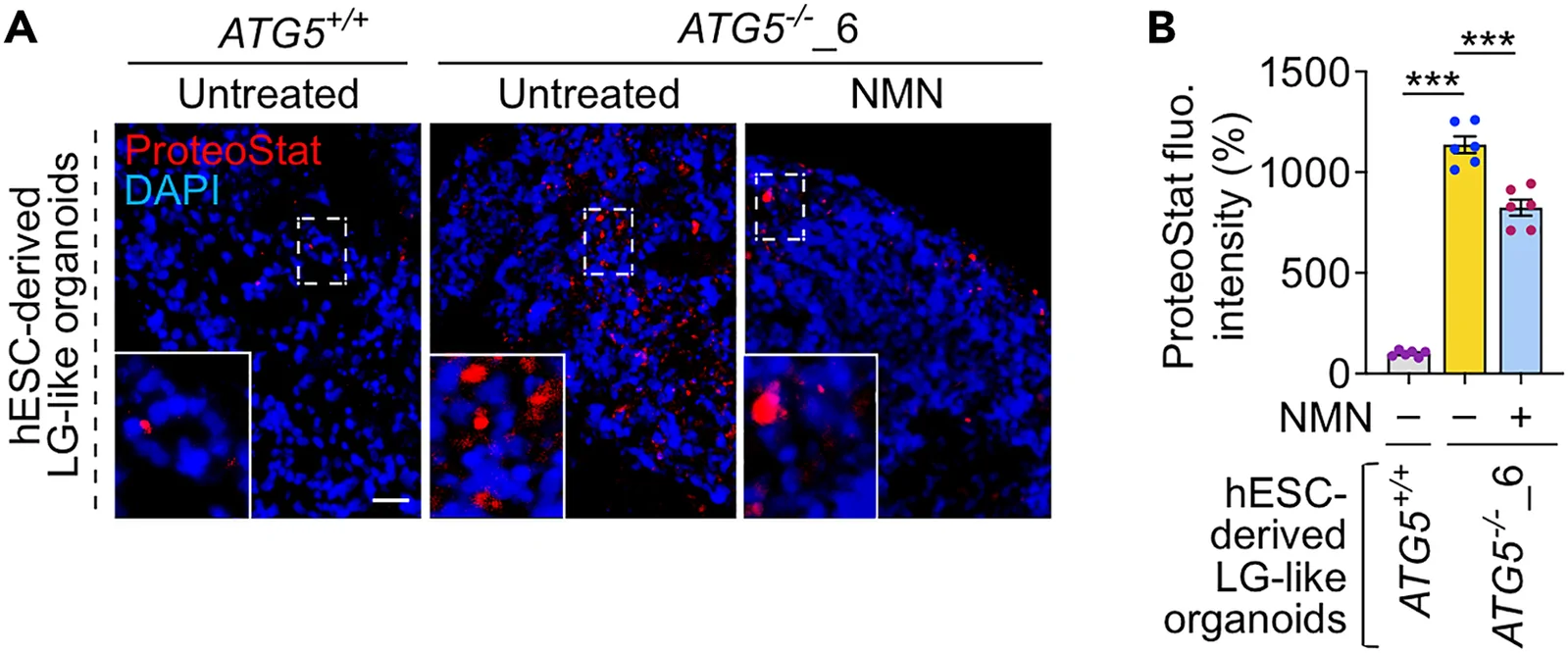
Accumulation of aggresomes in autophagy-deficient hESC-derived LG-like organoids is lowered by NMN treatment (A and B) Fluorescence images of ProteoStat staining (A) and quantification of ProteoStat fluorescence intensity (B) in *ATG5*^+/+^ and *ATG5*^−/−^_6 hESC-derived LG-like organoids, where *ATG5*^−/−^ organoids were treated with or without 1 mM NMN for 48 h. Graphical data is represented as mean ± SEM of n = 6 experimental replicates from 3 independent experiments (B). *P* values are calculated by one-way ANOVA followed by multiple comparisons with a two-stage linear step-up procedure of Benjamini, Krieger and Yekutieli. ∗∗∗P<0.001. Scale bar, 50 μm (A).

## Expected outcomes

Basal autophagy is not only critical for gland tissue development and functionality but also essential for the maintenance of cellular homeostasis and survival.^1^^,^^2^^,^^8^^,^^13^^,^^14^^,^^15^ Autophagy also mediates the clearance of aggregated proteins through a process called aggrephagy. Dysfunction of this degradative process leads to proteotoxic stress and accumulation of protein aggregates (aggresomes), as observed in autophagy-deficient cells and organoid models.^1^^,^^2^^,^^16^ The cellular consequences of autophagy deficiency, generated by knockout of *ATG5*, on cell death and aggresome accumulation can be assessed in *ATG5*^−/−^ hESC-derived LG-like organoids by respectively measuring TUNEL^+^ apoptotic nuclei and ProteoStat signals, as well as pharmacological rescue of these phenotypes following NMN treatment. Indeed, *ATG5*^−/−^ hESC-derived LG-like organoids displayed increased cell death and aggresome accumulation as evident from higher TUNEL^+^ apoptotic nuclei and ProteoStat signals, respectively, compared to the wild-type LG-like organoids (Figures 3A and 3B). As observed in *ATG5*^−/−^ hESC-derived neurons,^2^ NMN treatment reduced cell death (TUNEL^+^ apoptotic nuclei) and aggresome accumulation (ProteoStat signal) in *ATG5*^−/−^ hESC-derived LG-like organoids to levels comparable with *ATG5*^+/+^ LG-like organoids (Figures 4A and 4B).

Representative quantitative ranges for TUNEL and ProteoStat measurements are provided as a reference for expected outcomes. *ATG5*^+/+^ LG-like organoids (wild-type condition) typically showed low levels of cell death, with TUNEL^+^ apoptotic cells ranging from approximately 10%–20% (mean ± s.e.m. = 14.7 ± 1.14% as in Figure 3B). In contrast, *ATG5*^−/−^ LG-like organoids exhibited increased cell death, with TUNEL^+^ apoptotic cells ranging from approximately 30%–50% (mean ± s.e.m. = 42.3 ± 3.35% as in Figure 3B). Following NMN treatment, TUNEL^+^ apoptotic cells in *ATG5*^−/−^ LG-like organoids was reduced to the levels of the wild-type condition to approximately 10%–20% (mean ± s.e.m. = 14.0 ± 0.94% as in Figure 3B). ProteoStat fluorescence intensity was relatively low in *ATG5*^+/+^ LG-like organoids, which was fixed at 100%, whereas *ATG5*^−/−^ LG-like organoids displayed substantial elevation in ProteoStat signal, ranging from approximately 1000%–1300% (mean ± s.e.m. = 1119.1 ± 4.75% as in Figure 4B). NMN treatment modestly reduced ProteoStat fluorescence intensity in *ATG5*^−/−^ LG-like organoids, ranging from approximately 700%–900% (mean ± s.e.m. = 830.4 ± 37.89% as in Figure 4B). These values represent reference ranges obtained under the described imaging and analysis conditions and may vary depending on staining efficiency, microscope settings, and image-analysis parameters. The effects of NMN likely reflect its cytoprotective benefits in the context of autophagy deficiency.^2^^,^^8^ This protocol for assessing cellular phenotypes associated with autophagy-deficiency is expected to be applicable to other organoid models for studying the homeostatic role of autophagy, as well as to pharmacological interventions for therapeutic benefits.

## Limitations

This protocol addresses cellular deficits in LG-like organoids using an *ATG5*^−/−^ hESC model of autophagy deficiency. A limitation of this approach is the use of a single genetic autophagy-deficient hESC model, as loss of ATG5 may have other cellular consequences. Although additional genetic models would further strengthen the validation of autophagy-dependent phenotypes, additional autophagy-deficient hESC line is currently unavailable. In our original study, pharmacological inhibition of autophagy was used as a complementary approach to support the role of autophagy in LG-like organoid development.^1^ Organoids can lead to variability in cellular phenotypes and responses to pharmacological interventions. Incorporating microfluidic systems to better mimic tissue shear may help standardize organoid conditions and improve reproducibility.

## Troubleshooting

### Problem 1

Degradation of LG-like organoids.

### Possible solution

Ensure the use of an appropriate fixative and adequate fixing time. Carefully remove the surrounding Matrigel and confirm that the LG-like organoids are fully submerged in the fixative to achieve uniform fixation.

### Problem 2

The OCT compound-embedded frozen LG-like organoid block is challenging to section into smooth, flat sections.

### Possible solution

Lower the cryostat temperature and use a fresh area of the blade edge or replace the blade entirely. Ensure the OCT block is properly and uniformly frozen. Adjust the anti-roll plate and ensure it is correctly aligned.

### Problem 3

Detachment of LG-like organoid sections from the slides during the procedures.

### Possible solution

Use proper adhesive slides for strong and consistent attachment.

### Problem 4

Excessively high percentage of TUNEL-positive cells after TUNEL staining.

### Possible solution

Minimize tissue damage by carefully optimizing and validating the freezing and sectioning procedures.

### Problem 5

Uneven staining across the sections.

### Possible solution

Optimize the permeabilization time and staining volume to ensure complete coverage of the organoid section. Use a humidified chamber during incubation to prevent evaporation.

### Problem 6

Weak ProteoStat fluorescence signal after ProteoStat staining.

### Potential solution

Optimize the reagent concentration and dye dilution or increase the incubation time. Ensure the recommended incubation temperature and prepare fresh staining solution.

## Resource availability

### Lead contact

Further information and requests for resources and reagents should be directed to and will be fulfilled by the lead contact, Sovan Sarkar (s.sarkar@bham.ac.uk).

### Technical contact

Further technical information should be directed to and will be fulfilled by the technical contact, Gamze Kocak (g.kocak@bham.ac.uk).

### Materials availability

Further information and request for resources and reagents should be directed to the lead contact. The parental hESC line (WIBR3), originally generated and published by Rudolf Jaenisch lab at Whitehead Institute for Biomedical Research, was used in this study by Sovan Sarkar lab at the University of Birmingham under material transfer agreements, UBMTA 15–0593 and 15–0595. We are glad to share the hESC lines generated in this study with reasonable compensation by the requestor for their processing and shipping and a completed materials transfer agreement.

### Data and code availability

This protocol does not involve or generate any specific dataset or code.

## Acknowledgments

This work was supported by grants from the 10.13039/501100000265Medical Research Council (MRC) (MR/Z504488/1), 10.13039/501100000317Action Medical Research/10.13039/100012357LifeArc (GN3049), LifeArc (Philanthropic Fund P2019-0004, Pathfinder Award), 10.13039/100010269Wellcome Trust (109626/Z/15/Z), Wolfram Syndrome UK, and a Birmingham Fellowship to S.S., TÜBİTAK-BİDEB 2214A Fellowship to G.K. and S.S., YÖK 100/2000 PhD Scholarship, and TÜBİTAK-BİDEB 2211A Fellowship to G.K. S.S. is also a Former Fellow for life at Hughes Hall, University of Cambridge, UK.

## Author contributions

G.K. designed and performed the experiments and wrote the initial manuscript draft with inputs from S.S. G.K. prepared the figures. G.K. and S.S. acquired funding. G.K. and S.S. contributed to and approved the final manuscript.

## Declaration of interests

S.S. is a scientific advisor of NMN Bio Ltd, UK.
